# High-flow nasal cannula oxygenation reduces desaturation during percutaneous radiofrequency ablation under moderate sedation

**DOI:** 10.3389/fmed.2025.1517819

**Published:** 2025-07-23

**Authors:** Jiangling Wang, Qicheng Wu, Zewu Ding, Wen Zhang, Kangjie Xie, Xiaochun Mao, Xiangming Fang

**Affiliations:** ^1^Department of Anesthesia, Women's Hospital, Zhejiang University School of Medicine, Hangzhou, China; ^2^Department of Anesthesiology, Zhejiang Cancer Hospital, Hangzhou, Zhejiang, China; ^3^Department of Anesthesiology, The First Affiliated Hospital, Zhejiang University School of Medicine, Hangzhou, Zhejiang, China; ^4^Department of Thyroid Surgery, Zhejiang Cancer Hospital, Hangzhou, Zhejiang, China

**Keywords:** HFNC, desaturation, liver cancer, PRFA, moderate sedation, sedation, oxygen saturation

## Abstract

**Background:**

The majority of percutaneous radiofrequency ablation (PRFA) procedures for liver cancer are performed under ultrasound guidance and with moderate sedation. Oxygen desaturation is one of the most common and concerning adverse events that can be challenging to prevent during this procedure. High-flow nasal cannula (HFNC) oxygen therapy is effective in delivering high oxygen flow rates. This study aimed to evaluate the safety of HFNC oxygen therapy in preventing oxygen desaturation in patients undergoing ultrasound-guided PRFA with moderate sedation.

**Methods:**

In this prospective, randomized controlled study, 100 patients who underwent ultrasound-guided PRFA with moderate sedation were randomly divided into two groups: a low-flow oxygen group (6 L/min via an HFNC) and a high-flow oxygen group (40 L/min via an HFNC). The primary outcome was oxygen desaturation. Other adverse events were also recorded.

**Results:**

Patients who received high-flow oxygen (40 L/min) had a lower incidence of mild desaturation (0% vs. 6%, *p* = 0.24), moderate desaturation (4% vs. 30%; RR 7.5, 95% CI 2.07, 28.58, *p* = 0.0009), and severe desaturation (0% vs. 4%, *p* = 0.5) compared to those who received low-flow oxygen (6 L/min). Average oxygen saturation (SpO2) was significantly higher in the high-flow group (*p* < 0.0001). No significant differences were observed in other adverse events or perioperative variables.

**Conclusion:**

In patients undergoing ultrasound-guided PRFA with moderate sedation, high-flow oxygen therapy at 40 L/min through HFNC therapy resulted in higher average oxygen saturation levels and a reduced incidence of oxygen desaturation—particularly moderate and severe desaturation—compared to low-flow oxygen therapy at 6 L/min.

**Clinical trial registration:**

Identifier NCT05212064.

## Introduction

Liver cancer is the sixth most common cancer worldwide and the fourth leading cause of cancer-related deaths globally (1). By 2025, more than one million new cases of liver cancer are expected to be diagnosed annually (2). With advancements in techniques, early detection and treatment of liver cancer can lead to 5-year survival rates exceeding 70% (1, 3–8).

Percutaneous radiofrequency ablation (PRFA) under moderate sedation is recommended as the main non-surgical treatment for early-stage liver tumors (9–11) and tumors that are unsuitable for surgical resection or unresponsive to chemotherapy (12, 13). Due to central cardiorespiratory depression, complications caused by sedative-hypnotic drugs, such as respiratory depression, airway obstruction, and decreased compliance of the chest wall, often occur and are difficult to avoid fully (14–18). Hypoxemia and apnea frequently occur during sedation with traditional sedatives and techniques used in these procedures (19–22). Sedatives significantly increase the risk of desaturation and hypoxia, especially when combined with opioids (23). Therefore, airway management is critically important, as severe complications, including the risk of mortality, can arise if these complications are not detected in time (14, 16, 24).

The Optiflow HEALTHCARE device (Fisher and Paykel Healthcare, Panmure, Auckland, New Zealand) is a transnasal oxygen delivery system that provides humidified and warmed 100% oxygen at flow rates up to 60 L/min via a high-flow nasal cannula (HFNC) (25, 26). High-flow nasal cannula oxygen therapy can effectively decrease the incidence of desaturation and hypoxia during sedation (25, 27).

Adequate and stable oxygenation should be provided during sedation (28). Compared to regular nasal cannula oxygenation, high-flow nasal oxygen (HFNO) reduced the incidence of hypoxia from 21.2 to 2% during sedated gastrointestinal endoscopy in obese patients (29). In a study comparing preoxygenation via HFNO at 30–70 L/min with standard oxygen therapy at 10 L/min during bronchoscopy, the authors reported that HFNO provided better preoxygenation, as evidenced by significantly higher oxygen saturation levels (25). In another study comparing HFNO at 60 L/min with oxygenation via a Venturi mask immediately after extubation in patients who underwent major gynecological surgery in the Trendelenburg position, the authors found that the HFNO group had higher *PaO2/FiO2* ratios and less impaired gas exchange than the Venturi mask group (30). In addition, high-flow oxygen (70 L/min for 10 min) has been shown to increase apnea time in patients with difficult airways. The underlying mechanism involves rapid flushing of CO₂ from the nasopharyngeal dead space, generating 3–7 cmH₂O of positive end-expiratory pressure (PEEP), recruiting alveoli, and preventing atelectasis in the HFNO group (27).

Based on these previous studies, we hypothesize that HFNC oxygen therapy can reduce the incidence of desaturation in patients undergoing PRFA with moderate sedation.

## Materials and methods

### Study population

The study was approved by the ethics committee of Zhejiang Cancer Hospital (IRB-2022-262) and was registered on ClinicalTrials.gov (NCT05212064). This prospective, randomized clinical trial was conducted between 1 January 2022 and 17 August 2023. Written informed consent was obtained from all participants before the start of the study. The inclusion criteria were as follows: (1) aged 18–75 years old, (2) classified as American Society of Anesthesiologists (ASA) class I – III, and (3) undergoing percutaneous radiofrequency ablation. The exclusion criteria were as follows: (1) aged < 18 years or >75 years; (2) ASA class >III; (3) allergic to anesthetic solutions; (4) history of nosebleeds or coagulation disorders; (5) local infections (e.g., mouth, nose, or throat infection); (6) heart diseases such as congestive heart failure, severe aortic or mitral stenosis, cardiac surgery involving thoracotomy (e.g., coronary artery bypass graft or valve replacement) within the last 6 months, acute myocardial infarction within the last 6 months, or acute arrhythmia (including tachycardia and bradycardia) with hemodynamic instability; (7) chronic obstructive pulmonary disease (COPD) or other current acute or chronic lung diseases requiring supplemental continuous or intermittent oxygen therapy; (8) increased intracranial pressure; (9) fever, defined as a core body temperature >37.5 C; (10) severe anemia (30 g/L < hemoglobin <6 g/L); (11) emergency surgery; and (12) refusal to participate (15).

### Sample size and randomization

The incidence of desaturation during anesthesia with sedation has been reported to vary widely, ranging between 3.6 and 85% (31–33). Longhini et al. reported that HFNC therapy reduced the incidence of desaturation from 56 to 11% (34). Our institutional experience with hepatic radiofrequency ablation suggests a moderate desaturation rate of approximately 40%. Thus, the sample size was calculated based on an estimated reduction in the incidence of moderate desaturation from 40% in the control group to 10% in the HFNC group, with a = 0.05, power = 0.9, and an attrition rate of 20% (PASS Version 15.0.5). We determined that 100 patients would be needed for this study, with 50 patients in each group.

Randomization was performed using a computer-generated random sequence created with SAS PROC PLAN (version 9.3) at a 1:1 allocation ratio. Allocation concealment was rigorously maintained using sequentially numbered, opaque, and sealed envelopes. An independent statistician generated the sequence and prepared the envelopes. The circulating nurse opened the sequentially numbered envelope after the patient entered the operating room to reveal the allocation. The nurse then deployed the assigned Optiflow HEALTHCARE device. Both the statistician and the data recorders were double-blinded to the study. The data recorders were fully blinded, as the basic vital signs (SpO2, blood pressure, respiratory rate, and heart rate) were automatically recorded and stored in the monitor. The Modified Observer’s Assessment of Alertness/Sedation (MOAA/S) score was assessed every 3 min by the anesthesiologists. The data recorders were solely responsible for recording and were blinded to group allocation, and they had no patient care responsibilities.

However, the nurses and anesthesiologists could not be double-blinded, as they were responsible for managing complications and emergencies.

### Before anesthesia induction

All patients underwent 3-lead electrocardiography, pulse oximetry, and non-invasive blood pressure monitoring. The participants were randomly assigned to either the nasal cannula group or the HFNC group. All patients received oxygen via the Optiflow HEALTHCARE device (Fisher and Paykel Healthcare, Panmure, Auckland, New Zealand). The patients in both groups initially received oxygen at 6 L/min until they were sedated. The oxygen flow rate was subsequently increased to 40 L/min (37°C, 100% concentration) in the HFNC group, while the flow rate remained unchanged in the control group.

### Anesthesia induction and maintenance

Propofol was administered via target-controlled infusion (TCI), as previous research indicated that propofol consumption was significantly lower in the TCI group than in the manual bolus group. The effect-site concentration of propofol was initially set at 1.3 μg/mL and was then adjusted in a stepwise manner down to 0.3 μg/mL to maintain a sedation score between 3 and 4 (35). The depth of sedation was assessed and recorded every 3 min using the Modified Observer’s Assessment of Alertness/Sedation (MOAA/S) score. Oxycodone at a dose of 0.15 mg/kg (diluted to 1 mg/mL with normal saline; Mundipharma, Vantaa, Finland) was administered to both groups based on the findings from previous studies and experiences shared in our center. Rescue opioids were administered when the numerical rating scale (NRS) score was greater than 4 or when the patient had obvious, unwanted body movements. The rescue OXY dose was 0.05 mg/kg each time, with the total dose not exceeding 0.25 mg/kg (36). The PRFA procedure was started once the MOAA/S score was <4. The skin was anesthetized with 2% lidocaine before the ablation needle was inserted. The oxygen rate decreased to 6 L/min in the HFNC group after the procedure was completed, and the MOAA/S score was >4.

### Outcome measurement

The heart rate, respiratory rate, SpO_2_ (oxygen saturation measured by pulse oximetry), MOAA/S score, blood pressure, sedation, and ablation duration, total consumption of propofol and oxycodone, and NRS score were measured and recorded for each patient. When mild desaturation (90 ≤ SpO_2_ < 95%) occurred, the airway was opened using the jaw-thrust maneuver to improve oxygen desaturation. In the nasal cannula group, when moderate desaturation (75 ≤ SpO_2_ < 90% for < 60 s) occurred, the oxygen flow rate was increased to 10 L/min, and the jaw-thrust maneuver was performed. The definition of moderate desaturation was based on the recommendation of the World Society of Intravenous Anesthesia (SIVA) International Sedation Task Force (37) and aligned with thresholds used in prior clinical trials examining procedural sedation safety (15). The jaw-thrust maneuver was performed in the HFNC group only when mild desaturation occurred. When severe desaturation (75 ≤ SpO_2_ < 90% for ≥60 s or SpO_2_ < 75% for any longer duration) occurred, mask ventilation was performed. Emergency tracheal intubation was performed when deemed necessary, as determined by the anesthesiologist. All adverse events that occurred during the procedure, including those directly related to HFNC oxygen therapy, such as xerostomia, rhinalgia, pharyngalgia, headache, and barotrauma, were recorded until the patients were transferred to the ward (15). Adverse events were recorded using the 5-step tool proposed by the World Society of Intravenous Anesthesia (SIVA) International Sedation Task Force (37).

### Primary outcome

The primary outcome of this study was moderate desaturation (75 ≤ SpO_2_ < 90% for <60 s).

### Secondary outcomes

The secondary outcomes included mild desaturation (90 ≤ SpO_2_ < 95%), severe desaturation (75 ≤ SpO_2_ < 90% for ≥60 s or SpO_2_ < 75% for any longer duration), and the areas under the curve (AUCs) for heart rate, mean artery pressure (MAP), respiratory rate, and MOAA/S score during the procedure. The duration of surgery and anesthesia, anesthetic consumption, the highest NRS score, satisfaction scores of the anesthesiologists and surgeons, and sedation-and oxygen-related adverse events were also recorded and analyzed.

### Statistical analysis

Prism (version 9.0) was used for statistical analysis. Categorical variables were analyzed using Fisher’s exact test and presented as numbers (%) and relative risks (95% confidence intervals). Numerical variables with a normal distribution were analyzed using independent samples *t-*tests and presented as means (standard deviations). Numerical variables with a non-normal distribution were analyzed using the Mann–Whitney U test and presented as medians (minimums, maxima, or interquartile ranges). A *p*-value of <0.05 indicated statistical significance.

## Results

### Patient characteristics

**Figure 1:**
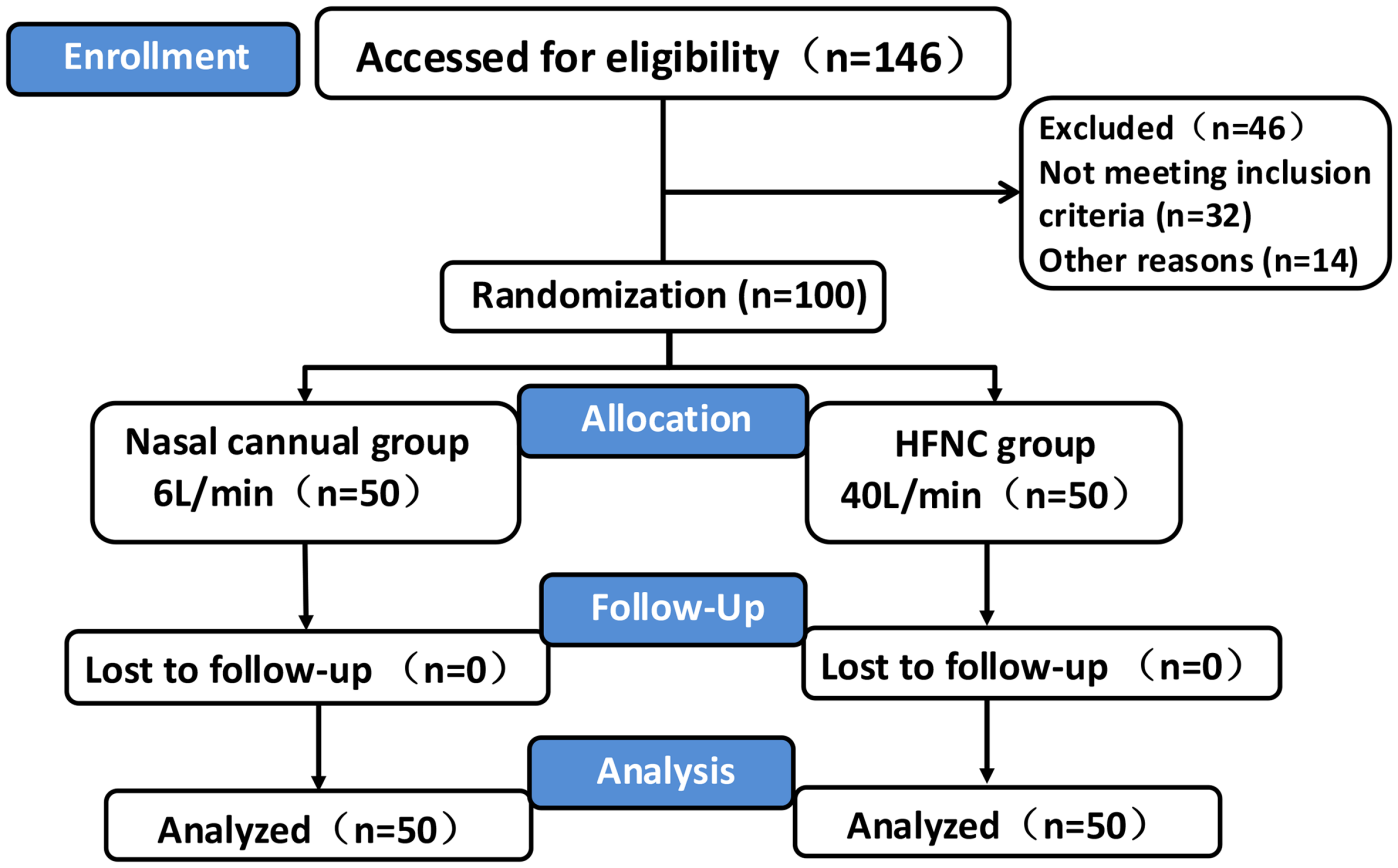
CONSORT flowchart. Flowchart depicting a clinical trial. Enrollment includes 146 participants, with 46 excluded. The 100 randomized participants are divided into two groups: nasal cannula (6 L/min, 50 participants) and high-flow nasal cannula (HFNC, 40 L/min, 50 participants). Both groups have no losses to follow-up, resulting in analysis of 50 participants from each group. PRFA percutaneous radiofrequency ablation, HFNC High-flow nasal cannula, TCI Target controlled infusion, PACU Post-anesthesia care unit.

The clinical data of the patients who participated in this study were included in the final analysis (Figure 1). The demographic characteristics, including age, sex, weight, BMI, and ASA physical status, were not significantly different between the two groups (Table 1).

**Table 1:** Demographic characteristics of the patients.

Variable	Control Group (*n* = 50)	HFNC Group (*n* = 50)	*P* value
Age (yrs.)	60.5 (9.4)	60.9 (9.3)	0.81
Male, no. (%)	33 (66)	32 (64)	>0.99
Weight (Kg)	64.5 (11.6)	67.6 (11.4)	0.20
BMI (Kg/m2)	22.9 (21.2, 25.9)	23.9 (22.2, 25.5)	0.28
ASA Physical Status I/II, no. (%)	39	44	0.29

There were no significant differences of the demographic characteristics between the two groups. The qualitative data are presented as no. (%), and the numerical data were presented as the mean (SD). HFNC, high-flow nasal cannula; SD, standard deviation; BMI, body mass index; ASA, The American Society of Anesthesiologists.

### Intraoperative characteristics

**Figure 2:**
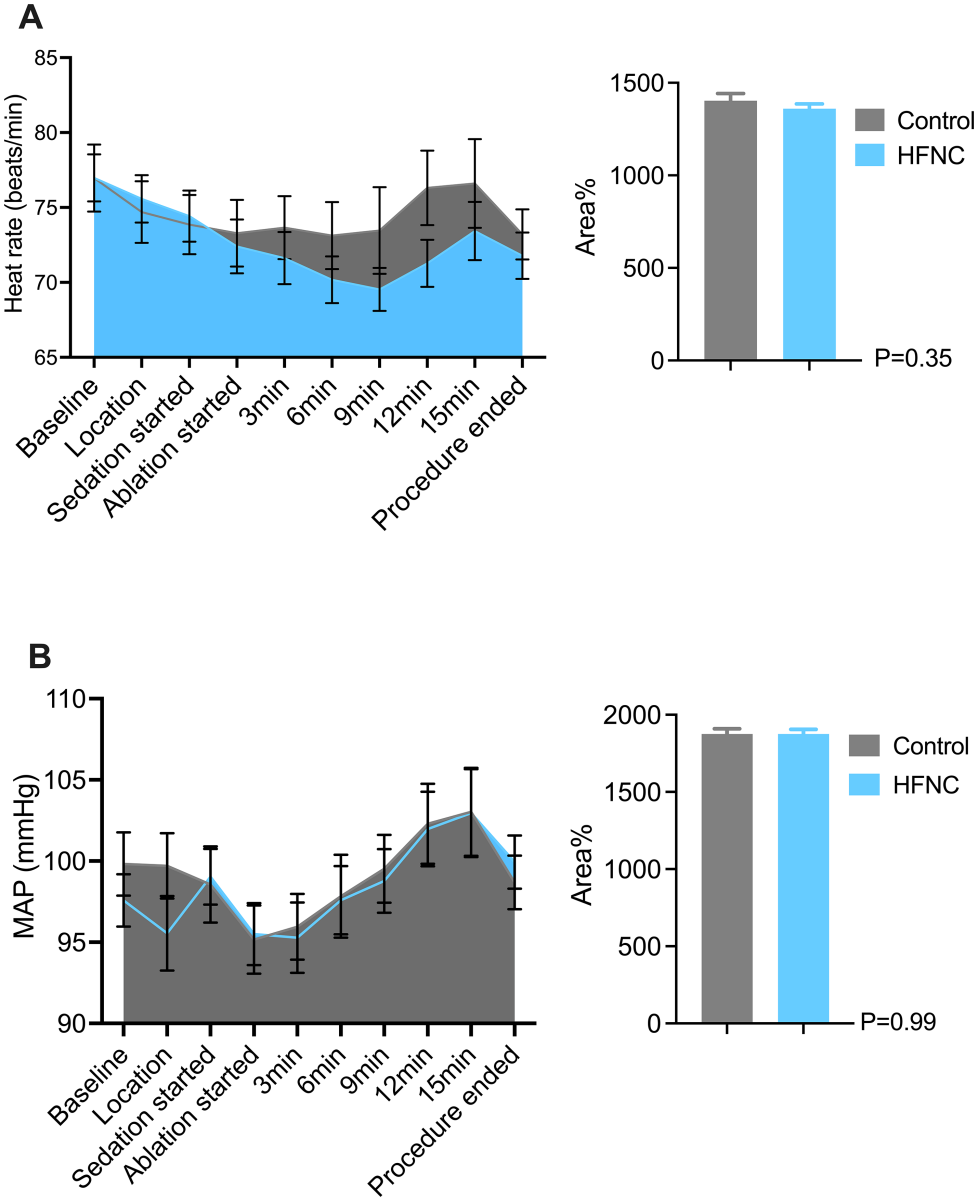
Two graphs comparing heart rate and mean arterial pressure (MAP) between control and HFNC groups during a procedure. Panel **A** shows heart rate changes over time, with areas representing control and HFNC. A bar graph shows no significant difference (P=0.35). Panel **B** depicts MAP changes, similarly colored, with its bar graph indicating no significant difference (P=0.99). Error bars represent variability.

**Figure 3:**
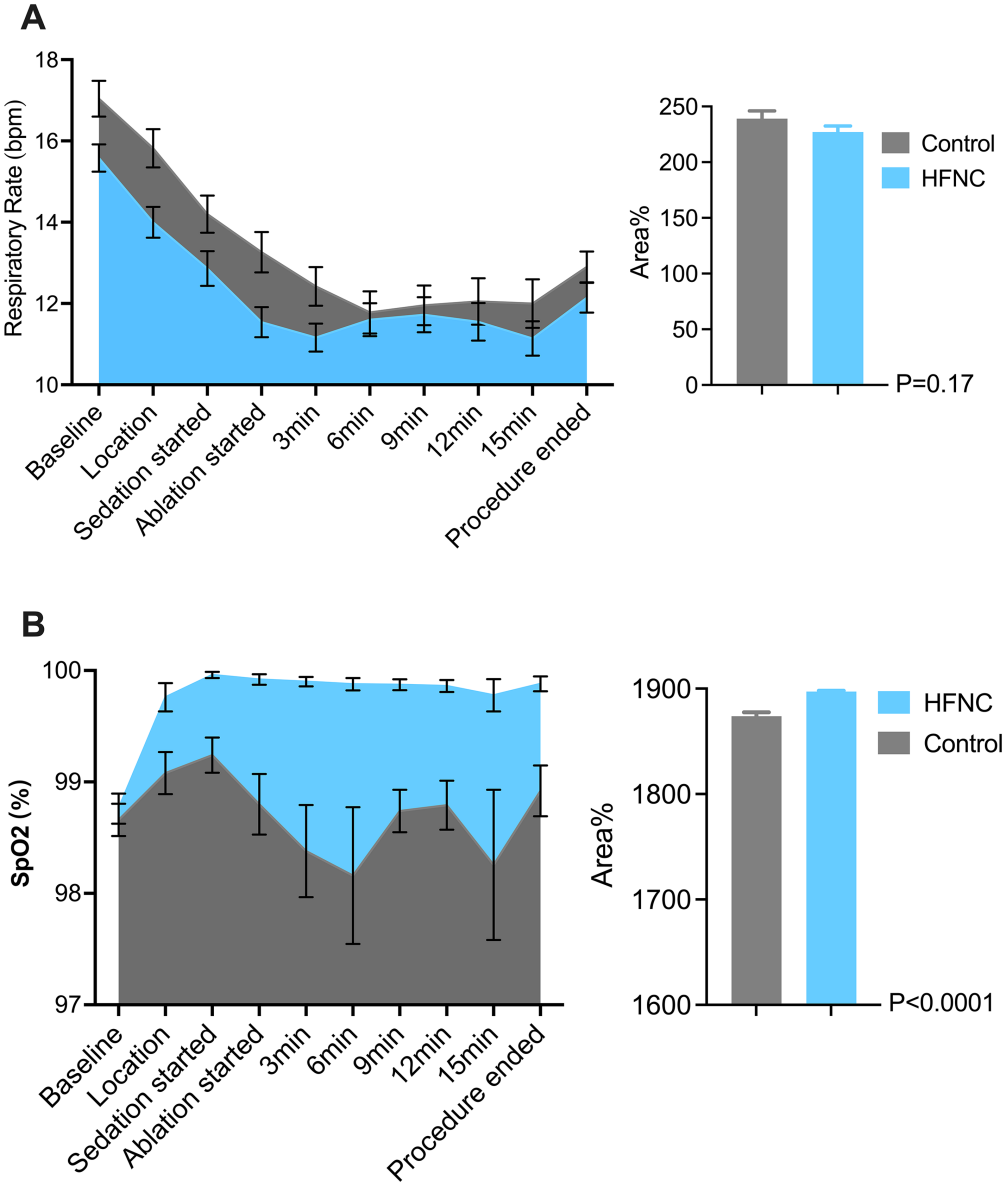
Two graphs compare respiratory data between Control and HFNC (High-Flow Nasal Cannula) groups. Graph **A** shows respiratory rate (bpm) over time during a medical procedure, with both groups starting high and decreasing, Control slightly higher. Graph **B** shows SpO_2_ percentage, mostly stable, with HFNC slightly above Control. Bar charts indicate area percentage differences with p-values: 0.17 for respiratory rate and less than 0.0001 for SpO_2_.

**Figure 4:**
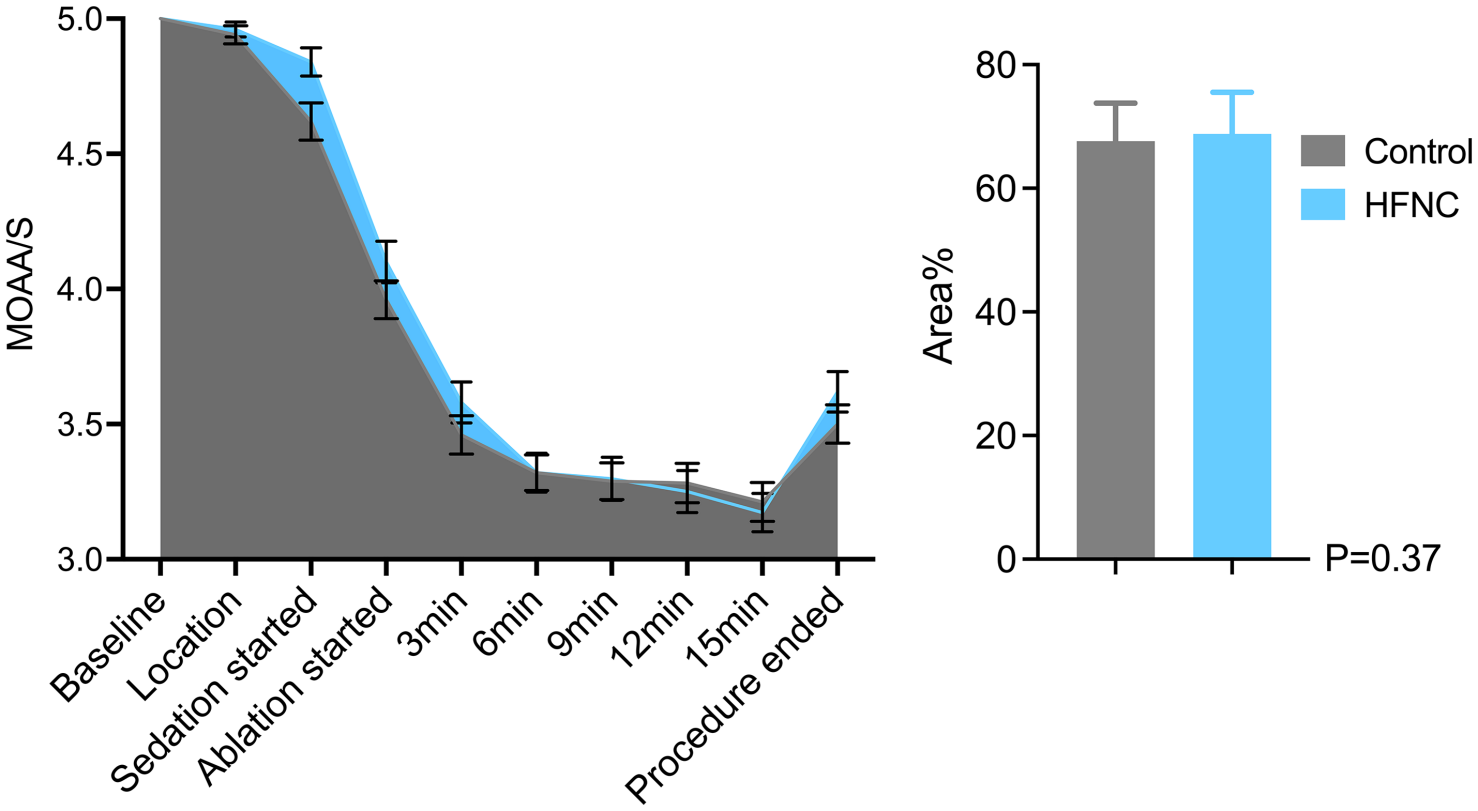
Line graph and bar chart comparing patient sedation levels during a medical procedure. The line graph shows Modified Observer’s Alertness/Sedation scale (MOAA/S) scores decreasing from baseline to ablation start and then stabilizing. The bar chart compares area percentages between control and HFNC groups, with similar values around seventy percent and a p-value of 0.37. Data were presented as mean and standard error of mean (SEM). The sky blue represents the HFNC group and the nickel grey represents the of nasal cannula group.

HFNC oxygen therapy decreased the incidence of moderate oxygen desaturation from 30 to 4%, which significantly differed between the two groups (*p* = 0.0009; RR, 7.5; 95% CI, 2.07–28.58). The incidences of mild desaturation and severe desaturation were 6 and 4%, respectively, in the control group. No patients in the HFNC group experienced mild desaturation or severe desaturation, with *p*-values of 0.24 and 0.50, respectively. The average SpO_2_ was 94% in the control group, with the lowest value being 67%. The average SpO_2_ was 99% in the HFNC group, with the lowest value being 84%. The duration of surgery and anesthesia, anesthetic consumption, adverse events, and satisfaction scores of the anesthesiologists and surgeons were not significantly different between the two groups (Table 2). Similarly, the AUCs for heart rate, MAP, respiratory rate, and MOAA/S score were not significantly different between the two groups (Figures 2–4).

**Table 2:** Intraoperative characteristics between the two groups.

Variable	Control group (*n* = 50)	HFNC group (*n* = 50)	Relative risk (95% CI)	*P* value
Time to target concentration	11.0 (8.0, 13.0)	11.0 (10.0, 12.0)		0.45
Surgery duration	29.5 (20.8, 40.5)	35.0 (24.8, 55.8)	–	0.16
Anesthesia duration	27.5 (19.8, 38.3)	35.5 (19.3, 50.5)	–	0.24
Propofol consumption (mg/kg/min)	0.05 (0.04, 0.08)	0.05 (0.04, 0.07)	–	0.48
Oxycodone consumption (mg/Kg)	0.15 (0.13, 0.17)	0.15 (0.14, 0.17)	–	0.6
Highest NRS	2.00 (1.00, 3.00)	2.00 (1.00, 3.00)	–	0.15
Mild oxygen desaturation, no. (%)	3 (6.0)	0 (0)	–	0.24
Moderate desaturation, no. (%)	15 (30.0)	2 (4.0)	7.5 (2.07, 28.58)	0.0009
Severe desaturation, no. (%)	2 (4.0)	0 (0)	–	0.50
Average SpO2	96 (67,100)	99 (84, 100)	–	<0.0001
Satisfaction score of anesthesiologist	10.0 (8.0, 10.0)	10.0 (9.0, 10.0)	–	0.33
Satisfaction score of surgeon	10.00 (8.8, 10.0)	10.0 (9.0, 10.0)	–	0.40

The incidence of the moderate desaturation was significantly lower and the average SpO2 was significant higher in the HFNC group. HFNC supportive oxygen therapy decrease mild, moderate and severe desaturation from 3% to 0, 15 to 2 and 4% to 0, respectively. Other characteristics were not significantly different between the two groups. The qualitative data are presented as no. (%) and relative risk (95% CI), and the numerical data were presented as the mean (SD). NRS, numerical rating scale; HFNC, high-flow nasal cannula; CI, confidence interval; SpO2, saturation of peripheral oxygen.

### Perioperative side effects and adverse events

Oxygen therapy-related adverse events and xeromycteria occurred in one patient in each group (*p* > 0.99), with an RR of 1.0 (95% CI, 0.11, 9.43). Rhinalgia occurred in one patient in the control group and two patients in the HFNC group (*p* > 0.99), with an RR of 0.50 (95% CI, 0.07, 3.71). These adverse events disappeared within minutes after recovery from anesthesia. In this study, patients did not experience other HFNC oxygen therapy-related adverse events, such as pharyngalgia, headache, and barotrauma. Mask ventilation was performed in two patients in the control group who experienced severe desaturation when their SpO_2_ further decreased. Their SpO_2_ levels then increased to above 95% within 1 min. No patient needed intubation in this study. Five patients in the control group and two patients in the HFNC group experienced hypertension, with a *p*-value of 0.44 and an RR of 2.5 (95% CI, 0.59, 10.85). In addition, two patients in the control group and no patients in the HFNC group experienced hypotension (*p* = 0.50). Four patients in the control group and two patients in the HFNC group experienced bradycardia (*p* = 0.68), with an RR of 2.0 (95% CI, 0.45, 9.07). The satisfaction scores of the anesthesiologists and surgeons did not significantly differ between the two groups, with *p*-values of 0.33 and 0.40, respectively (Table 3).

**Table 3:** Adverse effects between the two groups.

Variable	Control group (*n* = 50)	HFNC group (*n* = 50)	Relative risk (95% CI)	*P* value
Hypertension, no. (%)	5 (10.0)	2 (4.0)	2.5 (0.59, 10.85)	0.44
Hypotension, no. (%)	2 (4.0)	0 (0)	–	0.50
Bradycardia, no. (%)	4 (8.0)	2 (4.0)	2.0 (0.45, 9.07)	0.68
Mask Ventilation, no. (%)	2 (4.0)	0 (0)	–	0.50
Intubation, no. (%)	0 (0)	0 (0)	–	–
Xerostomia, no. (%)	1 (2.0)	1 (2.0)	1.00 (0.11, 9.43)	>0.99
Rhinalgia, no. (%)	1 (2.0)	2 (4.0)	0.50 (0.07, 3.71)	>0.99

Adverse effects were not significantly different between the two groups. The qualitative data are presented as no. (%) and relative risk (95% CI). HFNC, high-flow nasal cannula; CI, confidence interval.

## Discussion

Our study revealed that HFNC oxygen therapy at 40 L/min significantly reduced the incidence of desaturation from 40 to 4% and successfully prevented severe desaturation in the patients who underwent PRFA under moderate sedation. These findings align with prior evidence highlighting the efficacy of HFNC oxygen therapy in procedural sedation (28). For example, a recent study revealed that HFNC oxygenation can provide adequate and stable oxygenation and reduce the incidence of hypoxia from 21.2 to 2% during sedation (28, 29). This highly improves the safety of this procedure (Figure 3).

The mechanisms of desaturation during moderate sedation include hypoventilation, ventilation/perfusion (V/Q) mismatch, shunting, and diffusion impairment. Studies have shown that increasing FiO_2_ is an effective method to alleviate V/Q mismatch and diffusion impairment and prevent further shunting, whereas hypoventilation is most likely due to central respiratory depression caused by analgesics and sedatives (25). Preoxygenation with 100% oxygen via a mask is the traditional method for increasing oxygen reserves. However, this reserve is fixed once apnea begins (38). HFNC oxygen therapy is superior because it maintains oxygenation through three synergistic mechanisms: (1) Precise FiO₂ delivery (approximately 100% vs. 48–59% via nasal cannula), which directly addresses V/Q mismatch (39–42). (2) Dynamic CO₂ washout from the nasopharyngeal dead space, which reduces the risk of respiratory acidosis (27). (3) Positive airway pressure, which can reach up to as high as 7 cmH_2_O and has been shown to increase the duration of apnea in patients with difficult airways (27, 43, 44). The average SpO_2_ was 99% in the HFNC group and 96% in the control group, possibly because of the different FiO_2_ values between the two groups. The results of this study indicate that a high FiO_2_ value helps maintain SpO_2_ levels and prevent hypoxia during PRFA under moderate sedation. This study compared different oxygen concentrations, which may confound the interpretation of the results. The observed benefits may result from higher FiO2, enhanced flow delivery, or a combination of both factors.

Respiratory depression and desaturation are the most common adverse events that are not completely avoidable during sedation, especially when traditional analgesics are combined with opioids (19, 45), and the incidence of adverse events increases as the duration of surgery increases (14, 21, 24). Opioid consumption was not significantly different between the two groups; it was 0.15 (0.13, 0.17) mg/kg in the HFNC group and 0.15 (0.14, 0.17) mg/kg in the control group. The duration of PRFA varied depending on the location and size of the tumor and the surgical technique applied. However, the duration of the surgery exceeded 40 min for more than 30% of the patients, despite the fact that only two surgeons performed all the procedures in this study. The median duration of the procedure was 29.5 min in the control group and 35.0 min in the HFNC group. Oxycodone was used as analgesia more frequently than other opioids because of its lower propensity to cause respiratory depression (36). Target-controlled infusion of propofol was performed in this study, as it significantly reduced the total dose of propofol, shortened the recovery time, and provided a steady plasma concentration during moderate sedation (35, 46). The duration of surgery and anesthetic consumption were not significantly different between the two groups. Therefore, the differences in the incidences of desaturation between the two groups were not attributable to the different amounts of propofol and oxycodone administered.

In our study, we comprehensively evaluated the patients’ comfort and the tolerability of HFNC oxygen therapy, both of which are critical for its clinical adoption. Nasal dryness (xeromycteria) occurred in 2.4% (1/42) and 2.4% (1/41) of the patients in the HFNC and control groups, respectively. This condition resolved spontaneously within 15 min after the procedure. Nasal discomfort (rhinalgia) occurred in 4.8% (2/42) and 2.4% (1/41) of the patients in the HFNC and control groups, respectively. Serious adverse events, such as arrhythmia, cardiorespiratory arrest, permanent neurologic damage, and even death, can occur when desaturation is not detected and treated promptly (15, 24, 47).

The sedation-related mortality rate is 8/100000, which is similar to that associated with general anesthesia (47). A total of 40% of the patients in the control group exhibited desaturation, 6% of whom exhibited mild desaturation. No interventions were needed as SpO_2_ levels increased to above 95% within 10 s. The incidence of moderate desaturation was 30% in the control group and 4% in the HFNC group, and the jaw-thrust maneuver was performed when the oxygen flow was increased in the control group. In the HFNC group, only the jaw-thrust maneuver was performed, as the oxygen flow was already set at 40 L/min. SpO_2_ levels returned to normal within a short time in the majority of patients in both groups. Two patients in the control group had severe desaturation, and ventilation masks were used to prevent further decreases in SpO_2_ levels.

This study has several limitations. First, we did not measure the partial pressure of exhaled carbon dioxide (PETCO_2_), as the high-flow oxygen rate in the HFNC group made it difficult to measure this variable. Previous studies have reported increases in PETCO_2_ during conscious sedation (48). PETCO_2_ may have increased as the respiratory rates decreased in the present study. The respiratory rates were slightly lower in the HFNC group, but the areas under the curve were not significantly different between the two groups.

Second, SpO_2_, rather than partial pressure of oxygen (PaO_2_), was used to identify desaturation in this study. Previous studies have reported that SpO_2_ is more accurate when arterial saturation is above 90% and that its accuracy decreases when saturation falls below 90% (49–51). However, PaO2 provides a more accurate assessment of oxygenation. Nonetheless, as a convenient and non-invasive method for measuring oxygen saturation, SpO_2_ is still the gold standard for assessing oxygenation under sedation worldwide. More than 38% of the patients had moderate or severe desaturation in this study, which may increase the degree of bias and inaccuracy. Blood gas analysis would be preferable in future studies, as it provides information not only about PaO_2_ but also about PaCO_2_ and pH, which are more accurate than non-invasive methods.

Third, we did not include patients who were at high risk of desaturation, such as those with pulmonary disease. Moreover, we did not exclude overweight patients (BMI ≥ 25 kg/m^2^). However, the median BMI in the HFNC group was 23.9 (22.2, 25.5), which was higher than that in the control group (22.9 (21.2, 25.9)). There was only one overweight patient in the HFNC group, and three overweight patients in the control group had moderate desaturation. These results are consistent with recent findings indicating that increasing the airway pressure in obese patients helps prevent airway collapse (29) and hypoxemic respiratory failure (52). Our results suggest that HFNC oxygen therapy may decrease the incidence of desaturation in patients with high BMI, although studies specifically designed to confirm this finding are needed. Future investigations should prioritize the validation of HFNC therapy efficacy in populations with higher BMI (BMI ≥ 30 kg/m^2^), particularly given the mechanistic rationale for enhanced airway patency maintenance in obese patients under sedation.

Fourth, the sample size in this study was not large. More randomized controlled studies are needed to achieve significant differences between groups.

Fifth, a fundamental limitation is the confounding effect of oxygen concentration (FiO_2_). The high-flow group (40 L/min) inherently delivered a higher and more stable FiO_2_ compared to the low-flow group (6 L/min). Our study design did not allow us to isolate the specific effects attributable to high flow rates (e.g., dead space washout and positive airway pressure) from those primarily due to increased FiO_2_. Future studies addressing this question require control groups matched for FiO_2_ using devices such as Venturi masks or reservoir systems.

Sixth, the optimal oxygen flow rate for maximizing oxygenation during hepatic PRFA under sedation was not identified in this study. Future trials should incorporate dose–response designs to systematically evaluate the efficacy and safety of different flow rates (e.g., 10, 20, 30, and 40 L/min) to establish the most beneficial regimen.

Finally, despite these limitations, current evidence suggests that HFNC oxygen therapy is a practical option superior to conventional low-flow nasal cannulas for preventing hypoxemia during these procedures, without compromising the procedural workflow or patient safety. Key avenues for future research include studies with FiO2-matched controls, dose–response evaluations, and focused investigations in high-risk populations such as those with obesity or pulmonary comorbidities.

## Conclusion

In conclusion, HFNC oxygen therapy at 40 L/min increases the average oxygen saturation and reduces the incidence of desaturation compared to standard 6 L/min oxygen delivery during PRFA with moderate sedation. The incidence of respiratory adverse events significantly decreased as the incidence of moderate and severe desaturation decreased, thereby enhancing the safety of the entire procedure.

## Data Availability

The original contributions presented in the study are included in the article/supplementary material, further inquiries can be directed to the corresponding author.
